# Satisfaction With Health Care Services at the Pediatric Specialist Clinic of the National Referral Center in Malaysia: Cross-sectional Study of Caregivers’ Perspectives

**DOI:** 10.2196/33025

**Published:** 2022-05-25

**Authors:** Thinakaran M Selvarajah, Eiko Yamamoto, Yu Mon Saw, Tetsuyoshi Kariya, Nobuyuki Hamajima

**Affiliations:** 1 Department of Healthcare Administration Nagoya University Graduate School of Medicine Nagoya Japan; 2 Hospital Putrajaya Ministry of Health Putrajaya Malaysia; 3 Nagoya University Asian Satellite Campuses Institute Nagoya Japan

**Keywords:** pediatrics, caregivers, health care services, public hospital, Malaysia, public-private-partnership, children

## Abstract

**Background:**

The concept of customer satisfaction is gaining hold in all corporate sectors worldwide, and a satisfaction survey is used as a tool to discover service problems and as a chance for customers to rate their experience with health care services. A high degree of patient satisfaction with the services given has been found in numerous studies conducted in Malaysian public health care facilities. However, there is limited information available on caregiver satisfaction with pediatric clinics run by the Ministry of Health (MoH) of Malaysia.

**Objective:**

This was the first research performed at a public hospital’s pediatric clinic, which was the first hospital to adopt the public-private-partnership model under the MoH, with the aim of discovering the prevalence and factors affecting the satisfaction of caregivers at the national referral center.

**Methods:**

Cross-sectional research using the standard self-administered SERVQUAL questionnaire was conducted among caregivers accompanying their children to the clinic. The questionnaire consists of 16 paired statements to evaluate their expectations and experiences with the clinic services.

**Results:**

A total of 459 caregivers were involved in this study with a majority aged between 30 and 39 years (n=254, 55.4%). Caregivers from the Indian community (adjusted odds ratio [AOR] 2.91, 95% CI 1.37-6.18) and lower income groups (AOR 2.94, 95% CI 1.87-4.64), and those with lower educational backgrounds (AOR 3.58, 95% CI 1.19-10.72) were more likely to be satisfied with the quality of pediatric clinic services. Housewives/househusbands (AOR 0.48, 95% CI 0.25-0.90), on the other hand, appeared less likely to be satisfied with the services provided during their visit to the clinic. Looking at overall patient satisfaction, 50.5% (n=232) of caregivers demonstrated satisfaction with the quality of services, compared to 49.5% (n=227) of dissatisfied respondents.

**Conclusions:**

This paper suggests that, although most caregivers are satisfied with the services, greater emphasis must be placed on delivering reliable service in response to the MoH’s mission to provide quality and integrated people-centered health services in Malaysia.

## Introduction

Consumer satisfaction plays an increasingly important role in reforming health care quality and delivery in general across the United States and Europe [1]. The Integrated People-Centered Health Services is a global strategy by the World Health Organization that proposes a vision focused on providing people-centered and integrated health care services. This is a vision described as: “A future in which all people have access to health services that are provided in a way that responds to their personal preferences, are coordinated around their needs and are safe, effective, timely, efficient and of an acceptable quality, throughout their life course” [2]. Quality health care, in part, means meeting the needs of patients [3]. As the main stakeholders in a health care system, patients’ satisfaction reflects the expectations and general experience of health care services provided to them [4].

Patient satisfaction serves as an objective indicator of experiences, health outcomes, and trust with the health care system, representing whether the care provided has satisfied the patient’s needs and expectations [5]. Besides, it is an evaluation of the services provided by health care providers, which are influenced by both the level of expectations and the patient’s experience [6]. It is also possible to monitor the quality of care that could pave ways toward improving health care delivery [7]. Research suggests that satisfied patients are more prepared to seek medical guidance, comply with therapies, fulfill appointments, and refer other patients to a physician [8,9]. Research carried out in India indicates that surveys of patient satisfaction also act to hold doctors responsible [10]. In addition, the advent of increased competitiveness in the health care sector has led to the development of facilities that are committed to meeting the needs of patients. Highly ranked institutions in terms of service quality have better customer retention, lower expenses for bringing in new customers, increased profitability, and higher customer satisfaction [11-14].

The Ministry of Health (MoH) of Malaysia began its quality assurance program in the 1980s and has implemented many initiatives to improve the quality of health care delivery and enhance customer satisfaction, which includes the Client’s Charter and the acculturation of corporate values among employees who are caring, professional, and exercise teamwork [15]. Malaysia has provided impressive health benefits for its population through low-cost health care funded primarily by general revenue and taxes collected by the federal government [16].

The government has continuously committed itself to health care equity and accessibility, with the public health sector financing almost 95% of the cost of treatment and subsequently providing access to health care for more than 90% of its population [17]. Malaysians are also granted free access to consultations, treatment, and medications, as both inpatients and outpatients, for a nominal registration fee of Malaysian ringgit (RM) 1.00 (US $0.33) in all public health care facilities in the country [18]. This long-standing public policy has instilled a sense of entitlement among Malaysians that health care services in Malaysia should be free or cost the very least [16].

Many studies conducted at public health care facilities in Malaysia have shown a high level of patient satisfaction with the services provided [19]. However, to our best knowledge, no studies have been conducted on caregivers’ satisfaction in MoH pediatric outpatient clinics or facilities. This study, therefore, aims to ascertain the prevalence and factors influencing satisfaction and to identify areas of dissatisfaction among caregivers at the Paediatric Specialist Clinic of Tunku Azizah Hospital. This newly established hospital is a tertiary facility and a national referral center for the pediatric and women population.

## Methods

### Participants

This cross-sectional study was conducted at the Tunku Azizah Hospital, Kuala Lumpur, Malaysia. Participants were caregivers to children seen with an appointment at the clinic. Exclusion criteria were foreign nationals, refusal to participate, cognitively unsound, caregivers who could not read, and patient visiting for the first time. Only participants who met the eligibility criteria and agreed to participate in the study were enrolled.

The minimum sample size required was 364, which was calculated using the Raosoft (2004) online sample size calculator with a 95% confidence level, 0.5 SD, margin of error (CI) of 5%, and population size of 6714 (the monthly patient average). A total of 600 questionnaires were distributed to the clinic, and we received 502 responses, giving a rate of 83.7%. Of these 502 responses, 43 were unusable and were excluded from this study, and the remaining 459 (91.4%) questionnaires were analyzed. Some 2238 patients were registered for an appointment at the clinic during this data collection period.

### Data Collection and Ethical Considerations

This study was conducted at the hospital’s Paediatric Specialist Clinic by convenience sampling using a self-administered structured questionnaire. Every third registering caregiver was identified and given the questionnaires after seeing the doctor and while waiting for the date of their next consultation. Upon completing the questionnaire, participants were instructed to put it into an enclosed envelope. The sealed envelope is then passed to the nurse at the clinic counter.

Participants were recruited for 7 working days from September 3 to 12, 2019, upon receiving approval from the Medical Research and Ethics Committee (Research registration number NMRR-19-2191-49475[IIR]; MREC approval reference KKM/NIHSEC/P19-1924[5]). The goals and advantages of the study were explained in a verbal and written form attached to the questionnaires. Participants were assured of confidentiality in their involvement and that this would not have an effect on their treatment. They were reassured that their personal data would not be stored or used in any way possible and that their responses would only be used to enhance healthcare services. Informed consent was obtained from parents or guardians who agreed to participate. Following this study, only the data collection, findings and conclusions of this research have been published and any personal information collected from the participants is subject to confidentiality.

The principal researcher and two nurses were responsible for this data collection. This was part of a hospital-level survey assessing satisfaction among caregivers attending the clinic using the SERVQUAL instrument. SERVQUAL was initially developed for use in the marketing industry [20]. The SERVQUAL model is also known as a gap analysis model and is the most excellent tool for evaluating the quality of services [21]. The analysis of gaps is based on the difference between service quality expectations and perception. It was modified, translated, and validated in line with the Malaysian health care setting [22].

There are nine dimensions in this SERVQUAL tool, which includes the five original characteristics: tangibles, reliability, responsiveness, assurance, and empathy. Service outcomes and three other dimensions were included, including the core values of the MoH corporate culture: caring service, teamwork, and professionalism [23]. The current SERVQUAL tool that is used by the MoH is phrased in two languages (Malay and English).

The first part of the survey, which addressed the demographics of the respondents, was modified to include demographics of pediatric patients visiting the clinics. Sociodemographic data included independent variables such as the caregiver’s age, gender, race, marital status, education, employment sector, and household income level. These followed by age and gender of the child (patient), relationship with the caregiver, subspecialty that is being visited, waiting time, and the main problem encountered at the clinic during their visit.

The second section included the SERVQUAL tool, which contains 16 statements related to the respondents’ expectations on quality of service and 18 statements concerning their perception (experience) with the quality of service delivered. A 5-point Likert scale was used, ranging from “strongly disagree” (1) to “strongly agree” (5), with no verbal labels for scale points 2 through 4.

### Statistical Analysis

The data were coded, entered in Excel (Microsoft Corporation), and analyzed using SPSS version 21 (IBM Corp). Primary data on 459 responses were analyzed to examine satisfaction with services provided at the clinic. Sociodemographic characteristics of caregivers and patients and patient’s clinic visits were analyzed using descriptive analysis.

Each component from the satisfaction questionnaire was analyzed using a chi-square test. To describe caregivers’ and patients’ demographic profiles, a descriptive model with frequencies and percentages were developed. The median score of expectations and perceptions of caregivers and the mean gap scores for 16 paired items were evaluated. The difference in the mean values of perception and expectation for each component determined the caregiver’s satisfaction. This methodology assesses service quality by measuring the discrepancy (gap) between caregivers’ perceptions and expectations (service quality = P – E). “P” reflects the perception of the caregivers, and “E” refers to expectations of service delivery before encountering the actual service [24,25]. If the difference is negative, then there is dissatisfaction. To evaluate the mean satisfaction gap for each dimension, the mean gaps from all statements pertaining to a dimension is summed and then divided by the number of statements in that dimension.

Scores of four and five were considered to be satisfied, and the percentages were determined, while the other scores were considered to be dissatisfactory for the expectations and perception components. The Wilcoxon signed ranked test was used to make a comparison of distributions of expectations and perceptions. A logistic regression model was used to estimate the odds ratio (OR) and 95% CI for overall satisfaction level. The mean was computed for all gap scores of 16 paired statements, and an average of zero and higher is considered satisfied. *P*=.04 was considered to be statistically significant.

## Results

The sociodemographic characteristics of respondents and patients are summarized in Table 1. The whole study population was made up of 144 men and 315 women, with a substantial number of those aged 30 to 39 years (n=254, 55.4%). A total of 343 (74.7%) of the 459 respondents were Malays, while 408 (88.9%) of the total participants were married. A total of 231 (50.3%) of the respondents had completed a tertiary education, while 136 (29.6%) worked in the private sector. It is also worth noting that respondents with a lower household income account for more than half of the total or 266 (57.9%). Parents made up 432 (94.1%) of the caregivers who took part, and 236 (52.4%) of the patients were younger than 60 months.

Table 2 shows the characteristics of patient clinic visits, demonstrating that 211 (46%) of them had four or more appointments with the clinic. A total of 304 (66.2%) patients were seen by pediatric medical subspecialties, whereas 230 (50.1%) patients were seen in less than 60 minutes.

**Table 1:** Sociodemographic characteristics of caregivers and patients (N=459).

Characteristics		Male (n=144), n (%)	Female (n=315), n (%)	Total (N=459), n (%)
**Age (years)**				
	18-29	20 (13.9)	55 (17.5)	75 (16.3)
	30-39	71 (49.3)	183 (58.1)	254 (55.4)
	≥40	53 (36.8)	77 (24.4)	130 (28.3)
**Race**				
	Malay	107 (74.3)	236 (74.9)	343 (74.7)
	Chinese	16 (11.1)	39 (12.4)	55 (12.0)
	Indian	17 (11.8)	27 (8.6)	44 (9.6)
	Others	4 (2.8)	13 (4.1)	17 (3.7)
**Marital status**				
	Single	7 (4.9)	19 (6.0)	26 (5.7)
	Married	135 (93.7)	273 (86.7)	408 (88.9)
	Divorced/widowed	2 (1.4)	23 (7.3)	25 (5.4)
**Education background**				
	No formal education	1 (0.7)	3 (1.0)	4 (0.9)
	Primary education	6 (4.2)	13 (4.1)	19 (4.1)
	Secondary education	68 (47.2)	137 (43.5)	205 (44.7)
	Tertiary education	69 (47.9)	162 (51.4)	231 (50.3)
**Occupation sector**				
	Public sector	48 (33.3)	78 (24.7)	126 (27.5)
	Private sector	55 (38.2)	81 (25.7)	136 (29.6)
	Self-employed	26 (18.1)	33 (10.5)	59 (12.8)
	Housewife/househusband	5 (3.5)	84 (26.7)	89 (19.4)
	Others	10 (6.9)	39 (12.4)	49 (10.7)
**Household income^a,b^ RM^c^**				
	<3852	72 (50.0)	194 (61.6)	266 (57.9)
	3852-8319	58 (40.3)	104 (33.0)	162 (35.3)
	≥8320	14 (9.7)	17 (5.4)	31 (6.8)
**Relationship with patient**				
	Parents	136 (94.4)	296 (94.0)	432 (94.1)
	Others	8 (5.6)	19 (6.0)	27 (5.9)
**Patient’s age (months)**		272 (100.0)	187 (100.0)	459 (100.0)
	<60	137 (50.4)	99 (52.9)	236 (51.4)
	60-119	86 (31.6)	41 (21.9)	127 (27.7)
	120-179	37 (13.6)	37 (19.8)	74 (16.1)
	≥180	12 (4.4)	10 (5.4)	22 (4.8)

^a^Based on the Household Expenditure Survey (2014) published by the Malaysian Department of Statistics.

^b^US $1=RM 4.23

^c^RM: Malaysian ringgit.

**Table 2:** Characteristics of patient’s clinic visit (N=459).

Characteristics		Male (n=272), n (%)	Female (n=187), n (%)	Total (N=459), n (%)
**Frequency of visit**				
	2	101 (37.1)	73 (39.0)	174 (37.9)
	3	51 (18.8)	23 (12.3)	74 (16.1)
	≥4	120 (44.1)	91 (48.7)	211 (46.0)
**Subspecialty visited**				
	Pediatric medical	173 (63.6)	131 (70.1)	304 (66.2)
	Pediatric surgical	99 (36.4)	56 (29.9)	155 (33.8)
**Waiting time (minutes)**				
	<60	138 (50.7)	92 (49.2)	230 (50.1)
	60-119	61 (22.4)	38 (20.3)	99 (21.6)
	120-179	50 (18.4)	44 (23.5)	94 (20.5)
	≥180	23 (8.5)	13 (7.0)	36 (7.8)

Table 3 shows a comparison of expectation, perception, and satisfaction for each statement. This analysis shows that the respondents had a very high expectation for “staff politeness” (Q8), which was followed by “staff competency” (Q7) and “cleanliness of public toilets” (Q15). The lowest expectation was given for the “visual appeal of facilities” (Q2). However, it is interesting to note that the perception score fared slightly better for this statement. In terms of perception, the caregivers had the best experience with “staff politeness” (Q8), “staff work discipline” (Q13), and “cleanliness of public toilets” (Q15). On the contrary, the perception score was the lowest for “staff providing services at promised time” (Q3) and “appropriate waiting time” (Q16). The highest satisfaction gap was observed with the “appropriate waiting time” (Q16) followed by the “staff providing services at the promised time” (Q3), and the lowest satisfaction gap was for the statement “visually appropriate physical facilities” (Q2).

Table 4 depicts a comparison of expectation, perception, and satisfaction for each dimension. The “outcome” dimension had the most expectation from the caregivers, then by the “assurance” dimension. The lowest expectation was scored for the “caring service” dimension. The caregivers’ perception was highest for the “outcome” dimension as well. The “reliability” dimension had the lowest perception score and the widest satisfaction gap. The “tangibles” dimension, on the other hand, had the smallest satisfaction gap.

Crude and adjusted ORs (AORs) with 95% CIs of the factors associated with the overall satisfaction of caregivers with the quality of services provided are demonstrated in Table 5. The OR was adjusted to the 11 factors listed in Table 5. Caregivers from the Indian community (AOR 2.91, 95% CI 1.37-6.18) and lower household income groups (AOR 2.94, 95% CI 1.87-4.64) were approximately three times more likely to express higher levels of satisfaction with pediatric clinic service quality. Besides, respondents from lower educational backgrounds (AOR 3.58, 95% CI 1.19-10.72) were almost four times as likely to be satisfied with the services they received. However, housewives/househusbands (AOR 0.48, 95% CI 0.25-0.90) seemed less likely to be satisfied with the services provided during their visit to the clinic.

Looking into the overall satisfaction of the patients with the quality of service encountered at the Paediatric Specialist Clinic, it can be derived that 50.5% (n=232) of caregivers demonstrated satisfaction with the quality of services, as opposed to 49.5% (n=227) of the respondents being unsatisfied.

**Table 3:** Comparison of distribution for expectation, perception, and satisfaction for each statement.

Measurement statements^a^	Expectation		Perception		Satisfaction gap, mean (95% CI)	*Z* statistic^b^	*P* value^b^
	Score 4-5^c^ (%)	Median (IQR)	Score 4-5^c^ (%)	Median (IQR)			
Q1	92.2	5 (1)	88.9	5 (1)	–0.21 (–0.27 to –0.14)	–6.08	<.001
Q2	89.8	5 (1)	90.0	5 (1)	–0.07 (–0.13 to 0.00)	–1.98	.049
Q3	92.6	5 (1)	78.2	4 (1)	–0.39 (–0.48 to –0.30)	–8.29	<.001
Q4	93.7	5 (1)	87.8	4 (1)	–0.26 (–0.33 to –0.19)	–6.94	<.001
Q5	93.0	5 (1)	81.3	4 (1)	–0.36 (–0.44 to –0.28)	–7.88	<.001
Q6	93.7	5 (1)	89.3	5 (1)	–0.21 (–0.27 to –0.14)	–5.84	<.001
Q7	95.0	5 (1)	89.1	5 (1)	–0.26 (–0.33 to –0.19)	–6.88	<.001
Q8	95.2	5 (1)	91.3	5 (1)	–0.21 (­–0.28 to –0.15)	–6.36	<.001
Q9	93.2	5 (1)	88.0	4 (1)	–0.27 (–0.34 to –0.19)	–6.68	<.001
Q10	92.4	5 (1)	88.7	4 (1)	–0.23 (–0.31 to –0.16)	–6.27	<.001
Q11	94.3	5 (1)	89.8	5 (1)	–0.24 (–0.31 to –0.17)	–6.53	<.001
Q12	93.7	5 (1)	89.3	5 (1)	–0.22 (–0.29 to –0.15)	–6.12	<.001
Q13	94.8	5 (1)	90.4	5 (1)	–0.24 (–0.30 to –0.17)	–6.83	<.001
Q14	94.3	5 (1)	88.7	4 (1)	–0.27 (–0.34 to –0.20)	–7.26	<.001
Q15	95.0	5 (1)	90.4	5 (1)	–0.24 (–0.31 to –0.17)	–6.44	<.001
Q16	92.8	5 (1)	79.3	4 (1)	–0.48 (–0.57 to –0.39)	–9.35	<.001

^a^Refer to Multimedia Appendix 1 for measurement statements.

^b^Wilcoxon signed rank test.

^c^Caregivers scoring 4 and 5.

**Table 4:** Comparison of distribution for expectation, perception, and satisfaction for each dimension.

SERVQUAL dimensions^a^	Expectation		Perception		Satisfaction gap, mean (95% CI)	*Z* statistic^b^	*P* value^b^
	Score 4-5^c^ (%)	Median (IQR)	Score 4-5^c^ (%)	Median (IQR)			
Tangibles	90.4	5.00 (0.67)	84.7	4.33 (1.00)	–0.17 (–0.22 to –0.12)	–6.15	<.001
Reliability	90.4	5.00 (0.67)	75.4	4.33 (1.00)	–0.35 (–0.44 to –0.30)	–9.69	<.001
Responsiveness	92.6	5.00 (1.00)	81.7	4.50 (1.00)	–0.28 (–0.35 to –0.21)	–7.58	<.001
Assurance	93.7	5.00 (0.67)	85.6	4.67 (1.00)	–0.25 (–0.31 to –0.19)	–8.07	<.001
Empathy	91.7	5.00 (1.00)	86.1	4.50 (1.00)	–0.25 (–0.32 to –0.18)	–6.96	<.001
Outcome	94.3	5.00 (1.00)	89.8	5.00 (1.00)	–0.24 (–0.31 to –0.17)	–6.53	<.001
Caring service	89.1	5.00 (0.71)	77.8	4.43 (1.00)	–0.27 (–0.33 to –0.22)	–8.73	<.001
Teamwork	92.6	5.00 (1.00)	86.3	4.50 (1.00)	–0.24 (–0.30 to –0.18)	–7.32	<.001
Professionalism	90.6	5.00 (0.75)	77.6	4.50 (1.00)	–0.27 (–0.33 to –0.21)	–8.87	<.001

^a^Refer to Multimedia Appendix 1 for dimension statements.

^b^Wilcoxon signed rank test.

^c^Caregivers scoring 4 and 5.

**Table 5:** The odds ratio (OR) and 95% CI of factors associated with the level of caregiver’s satisfaction (N=459).

Characteristics		Crude OR (95% CI)	Adjusted OR^a^ (95% CI)	*P* value
**Gender**				
	Male	1 (reference)	1 (reference)	N/A^b^
	Female	1.21 (0.82-1.80)	1.41 (0.89-2.21)	.14
**Age (years)**				
	18-29	1 (reference)	1 (reference)	N/A
	30-39	1.19 (0.71-1.99)	1.51 (0.85-2.69)	.16
	≥40	1.02 (0.58-1.80)	1.45 (0.74-2.82)	.28
**Race**				
	Malay	1 (reference)	1 (reference)	N/A
	Chinese	0.70 (0.39-1.25)	0.69 (0.37-1.29)	.25
	Indian	2.81 (1.40-5.64)	2.91 (1.37-6.18)	.005
	Others	1.93 (0.70-5.34)	1.67 (0.56-4.97)	.36
**Education background**				
	High education	1 (reference)	1 (reference)	N/A
	Low education	3.74 (1.36-10.24)	3.58 (1.19-10.72)	.02
**Occupation sector**				
	Public sector	1 (reference)	1 (reference)	N/A
	Private sector	1.24 (0.76-2.02)	1.00 (0.59-1.69)	.98
	Self-employed	1.66 (0.89-3.10)	1.23 (0.62-2.46)	.56
	Housewife/househusband	0.93 (0.54-1.60)	0.48 (0.26-0.92)	.03
	Others	1.39 (0.72-2.70)	0.71 (0.33-1.56)	.40
**Household income**				
	Medium income	1 (reference)	1 (reference)	N/A
	Low income	2.71 (1.81-4.06)	2.94 (1.87-4.64)	<.001
	High income	1.48 (0.68-3.21)	1.51 (0.67-3.39)	.32
**Frequency of visit**				
	2	1 (reference)	1 (reference)	N/A
	3	1.27 (0.74-2.20)	1.22 (0.67-2.20)	.52
	≥4	1.01 (0.68-1.51)	1.14 (0.73-1.78)	.57
**Subspecialty visited**				
	Pediatric medical	1 (reference)	1 (reference)	N/A
	Pediatric surgical	0.95 (0.64-1.40)	0.94 (0.61-1.46)	.80
**Waiting time (minutes)**				
	<60	1 (reference)	1 (reference)	N/A
	60-179	0.59 (0.37-0.92)	0.63 (0.39-1.03)	.07
	≥180	0.66 (0.40-1.09)	0.67 (0.39-1.15)	.15
**Relationship with patient**				
	Others	1 (reference)	1 (reference)	N/A
	Parents	0.49 (0.22-0.12)	0.67 (0.26-1.72)	.40
**Patient’s age (months)**				
	<60	1 (reference)	1 (reference)	N/A
	60-179	0.98 (0.64-1.49)	0.83 (0.52-1.34)	.45
	≥180	0.78 (0.48-1.26)	0.64 (0.36-1.12)	.12

^a^Adjusted for 11 factors: gender, age, race, educational background, occupation sector, household income, frequency of visit, subspecialty visited, waiting time, relationship with patient, and patient’s age.

^b^N/A: not applicable.

## Discussion

Tunku Azizah Hospital is the first public-private-partnership (PPP) project in Malaysia under the MoH, using the private finance initiative (PFI) model. This facility was initially known as the Kuala Lumpur Women and Children Hospital but was renamed in January 2020 to commemorate the present Queen. The hospital started operations in phases from February 2019, and the Paediatric Specialist Clinic was the first to offer its services to the public. To our best knowledge, this is the first paper that discusses the factors affecting overall caregivers’ satisfaction and identifies areas of dissatisfaction in a pediatric clinic run by MoH in Malaysia.

As can be seen from the results of the analysis, there was a negative satisfaction gap in all dimensions, suggesting that none surpassed the expectations of the caregivers. This result is also consistent with another study carried out in Singapore [26] using a similar instrument. Negative gaps are commonly predicted, as expectations for optimum service are rarely met.

Overall, 50.5% (n=232) of caregivers were satisfied with the pediatric clinic and the quality of services provided during this study period. This result is in contrast with another study by Aniza et al [27] that was conducted at the Paediatric Clinics of the University of Kebangsaan Malaysia Medical Center that had a 90.5% satisfaction rate. Such a finding may be due to the higher expectations that caregivers had with a newly opened health care facility.

There was evidence that respondents with lower educational levels and household income, and those of the Indian community have better satisfaction with health care services at the clinic. Several authors have found that demographic characteristics, such as gender, age, and education, were strongly linked to respondent’s satisfaction. Although satisfaction levels were not significantly associated between age and gender in this study, their prevalence in other studies was significant, where males were found to be more satisfied than female respondents [28,29]. Another study indicated that gender did not have a significant impact on the satisfaction rate in their findings [30]. Even if age does not appear to be associated with satisfaction levels in some research [31], one study found that the average satisfaction rate improved with the increase in age and that the satisfaction rate was the most feasible with those older than 55 years [32].

This study found a statistically significant inverse association between the level of education and the satisfaction of caregivers, which is comparable to other studies, indicating that respondents who were less educated were more satisfied than those with higher education [33-35]. This result could be due to higher standards set by the educated group, as they believed they were more acquainted with the care they would obtain. Besides, those with higher education levels are pragmatic and able to see the services objectively, and they were dissatisfied when the level of services did not meet their expectation [24]. Similar to other research done, this study also shows that a lower income group has shown more satisfaction toward the services at the clinic [18]. This group of caregivers consisted of more than half of the total respondents and were more concerned about the costs associated with health care delivery. Thus, they were more satisfied with the services at this outpatient facility accessible at a low cost of RM 1.00 (US $0.33). In this study, caregivers from the Indian ethnic minority were more satisfied, as opposed to the other ethnic groups. This is different from another study that pointed out that the minority ethnicity reported lower satisfaction and less positive experiences with health care services [36].

One interesting observation from this study is that the housewives/househusbands had a relatively lower satisfaction level at the clinic, which is similar to another published study [37]. This could be due to the different commitments they have made, and they anticipate that the appointment will be completed in a short period of time.

In this research, all statements and dimensions revealed negative satisfaction scores indicating that none met the expectations of the caregivers. However, caregivers’ experiences from this study point out that the staff in the clinic had shown politeness and good work discipline, and that the public toilets were clean. Caregivers were the least satisfied with the waiting time and had concerns with services not being provided at the promised time. A study conducted in France also suggested dissatisfaction among patients with waiting times [38]. The MoH had a target of 90-minute waiting times. Nevertheless, almost half of the patients (median waiting time) were seen by doctors in less than 60 minutes or at an average of 83 minutes for all cases.

The respondents also pointed out better than expected experience with the visual appeal of the health care infrastructure. As this facility is a PPP project, it has integrated certain nonconventional elements into its architecture and design that reflect sociocultural, economic, professional, and aesthetic priorities. This reflects and reinforces contemporary concepts of patienthood and caring, and projects the implementation of patient-centeredness.

Caregivers’ satisfaction with services can be assessed based on the following service attributes as highlighted by Parasuraman et al [20]. The five original SERVQUAL dimensions are defined as reliability (the ability to perform the promised service dependably and accurately), responsiveness (willingness to help customers and provide prompt service), assurance (employees’ knowledge and courtesy, and their ability to inspire trust and confidence), empathy (caring, individualized attention given to customers), and tangibles (the appearance of physical facilities, personnel, and written materials). An additional four dimensions (service outcome, caring service, teamwork, and professionalism) were included in the MoH version of SERVQUAL.

Caregivers had the highest expectation for service outcomes, and they also had the best experience with the outcome of their visits to the outpatient clinic, which indicates that they were pleased with the consultations or treatments they got. However, the “reliability” dimension needs to be substantially enhanced, as this had the most substantial satisfaction gap. The care providers should focus on reducing the waiting time in the clinic and mobilizing resources to enhance customer satisfaction further. While the “tangibles” dimension had the lowest satisfaction gap over all other dimensions, it is equally important to clean, maintain, and gleam the building premises. Maintaining the building premises is essential to maintain the properties and protect the inhabitants of the building. Proper building maintenance ensures that the building and the environment remain secure, clean, and safe to function.

There were some limitations to this study. First, we carried out our study at a tertiary, national referral center run by consultants, trained specialists, and postgraduate trainees, which differs from those in primary public clinics, which are mostly run by medical officers without postgraduate qualifications. Therefore, the results of our study cannot be generalized to reflect the performance of other clinics in this region. Since the questionnaires used were self-administered, patients who were illiterate were not recruited. Besides, convenience sampling, while unavoidable, is another drawback to this research due to the high probability of bias in sampling. Hence, the findings may not be generalized to the broader population. Additionally, not all aspects of the services, such as pharmacy and prescription drugs, have been evaluated in this study. These factors have been found to influence patient satisfaction significantly [39,40]. This study was also carried out at a relatively new facility, which could have resulted in a positive satisfaction bias among some respondents.

We believe that future surveys with questionnaires should avoid using all positively expressed statements to assess service quality. It would mitigate the overall bias if there were a combination of positive and negative framed statements [41]. Additionally, other aspects of services, such as registration, pharmacy, and prescription drugs, should be considered to gauge the complete experience of caregivers while visiting health facilities. Value-driven outcome tools that measure quality and include both nationally accepted and validated measures, as well as local physician- and patient-defined outcome measures, should also be considered [42].

The purpose of this paper is to demonstrate how caregivers’ perspectives are influenced by various aspects of clinic services, as well as to assess the differences between what they expect and what they experience when engaging with a public health care facility. The study looked at data from a survey that measured caregiver opinions across several dimensions and found that the service outcome dimension was assigned the highest weight, and the pediatric clinic met expectations. In addition, respondents from lower income groups, Indian ethnicity, and those with less education were more appreciative of the services offered.

Regardless, consistent measures must be put in place to increase customer satisfaction, which will strengthen health care delivery standards. The incorporation of patient-centered care as a strategic investment goal, as well as the development and implementation of constructive, organized strategies that involve frontline clinicians in the process of improving caregiver satisfaction, will benefit hospital management. Routine satisfaction assessments should be conducted using improvised questionnaires or other tried-and-true methods to identify unsatisfactory domains that require substantial improvements. These measures will ensure that the services provided are in line with the MoH’s mission of providing quality integrated, people-centered health care to the masses. Future studies may be able to compare additional hospitals that use the PFI model, as well as provide more information about the variations discovered in this study.

## Appendix

Multimedia Appendix 1 Measurement statements and dimensions concerning the satisfaction questionnaire used in the study.

## Abbreviations

AOR: adjusted odds ratio
MoH: Ministry of Health
OR: odds ratio
PFI: private finance initiative
PPP: public-private-partnership
RM: Malaysian ringgit
