# Supplementary material for: Satisfaction With Health Care Services at the Pediatric Specialist Clinic of the National Referral Center in Malaysia: Cross-sectional Study of Caregivers’ Perspectives
Source: JMIRx Med. 2022 May 25;3(2):e33025. doi: 10.2196/33025 (PMC10414228; doi:10.2196/33025)
Supplement: Multimedia Appendix 1 [file xmed_v3i2e33025_app1.pdf]

Measurement statements and dimensions concerning satisfaction questionnaire used in the study.

Statements	
Q1	Up-to-date equipment
Q2	Visually appealing physical facilities
Q3	Staff provides services at the promised time
Q4	Staff render the services right, every time
Q5	Patients can expect prompt service from the staff
Q6	Staff always willing to help with sincere interest
Q7	Staff do their job competently
Q8	Staff are polite
Q9	Staff always understand patients' needs
Q10	Staff render patients personal attention
Q11	Staff provide effective treatment
Q12	Staff work as a team in rendering treatment
Q13	Staff display good work discipline
Q14	Staff are knowledgeable
Q15	Public toilets are always clean
Q16	Waiting time is appropriate
Q17	Staff provide services in accordance with the ministry's client charter
Q18	Overall level of satisfaction

Dimensions	
<u>SERVQUAL dimensions</u>	
Tangibles	Q1, Q2, Q15
Reliability	Q3, Q4, Q16
Responsiveness	Q5, Q6
Assurance	Q7, Q8, Q14
Empathy	Q9, Q10
Outcome	Q11
<u>Ministry of Health corporate culture core value dimensions</u>	
Caring service	Q3, Q4, Q5, Q6, Q8, Q9, Q10
Teamwork	Q7, Q12
Professionalism	Q3, Q4, Q7, Q9, Q10, Q11, Q13, Q14
